# Chinese Herbal Medicine for the Treatment of Depression: Effects on the Neuroendocrine-Immune Network

**DOI:** 10.3390/ph14010065

**Published:** 2021-01-14

**Authors:** Chan Li, Bishan Huang, Yuan-Wei Zhang

**Affiliations:** 1 School of Life Sciences, Guangzhou University, Guangzhou 510006, China; lichan@gzhu.edu.cn (C.L.); 2111914032@e.gzhu.edu.cn (B.H.); 2 Department of Psychiatry, School of Medicine Yale University, New Haven, CT 06511, USA; 3 Department of Pharmacology, School of Medicine Yale University, New Haven, CT 06511, USA

**Keywords:** Chinese herbal medicine, depression, neuroimmune system, neuroendocrine system, neuroendocrine-immune network, neuroinflammation, HPA axis

## Abstract

The neuroimmune and neuroendocrine systems are two critical biological systems in the pathogenesis of depression. Clinical and preclinical studies have demonstrated that the activation of the neuroinflammatory response of the immune system and hyperactivity of the hypothalamus–pituitary–adrenal (HPA) axis of the neuroendocrine system commonly coexist in patients with depression and that these two systems bidirectionally regulate one another through neural, immunological, and humoral intersystem interactions. The neuroendocrine-immune network poses difficulties associated with the development of antidepressant agents directed toward these biological systems for the effective treatment of depression. On the other hand, multidrug and multitarget Chinese Herbal Medicine (CHM) has great potential to assist in the development of novel medications for the systematic pharmacotherapy of depression. In this narrative essay, we conclusively analyze the mechanisms of action of CHM antidepressant constituents and formulas, specifically through the modulation of the neuroendocrine-immune network, by reviewing recent preclinical studies conducted using depressive animal models. Some CHM herbal constituents and formulas are highlighted as examples, and their mechanisms of action at both the molecular and systems levels are discussed. Furthermore, we discuss the crosstalk of these two biological systems and the systems pharmacology approach for understanding the system-wide mechanism of action of CHM on the neuroendocrine-immune network in depression treatment. The holistic, multidrug, and multitarget nature of CHM represents an excellent example of systems medicine in the effective treatment of depression.

## 1. Introduction

Depression is a persistent and recurring mental illness, affecting more than 264 million people of all ages worldwide. It is also a major contributor to the global burden [1] and a leading cause of elevated disability [2]. Depression is clinically characterized by repeated depressive episodes, including anhedonia, insomnia, decreased speech, loss of interest and enjoyment, helplessness, and decreased energy [3]. Most researchers view depression as a multigenetic and multifactorial syndrome, which results from the complicated interplay of environmental and genetic factors and presents comorbidity with other diseases [4].

**Figure 1:**
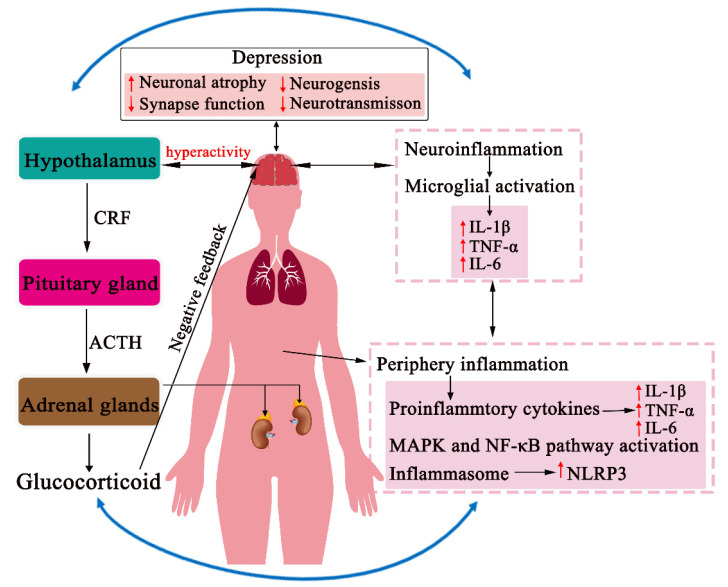
The neuroendocrine-immune network in the pathogenesis of depression. In response to stress, the peripheral or neuroimmune system activates the release of HPA axis hormones, whereas the stress-induced hyperactivation of the HPA axis also stimulates a proinflammatory or neuroinflammatory response. Intersystem crosstalk occurs at many levels through neural, immunological, and humoral interactions and subsequently results in the dysfunction of the central nervous system (CNS) in the pathogenesis of depression.

While current antidepressant medications, such as selective monoamine reuptake inhibitors and glutamate transmission-enhanced fast-acting antidepressants, can improve mental states of depression, these drugs are far from ideal, because they have severe side effects and low rates of efficacy [5]. Growing evidence suggests that central nervous system (CNS)-targeted medications alone are insufficient, and the development of novel medications or approaches for effective and systematic depression treatment is a pressing task [4,6]. In recent decades, many divergent biological systems have been identified to be involved in the pathogenesis of depression. In particular, studies have shown that the activation of the neuroinflammatory response of the immune system and hyperactivity of the hypothalamus–pituitary–adrenal (HPA) axis of the neuroendocrine system are two critical triggers in the etiology of depression [7] (Figure 1). It should be emphasized that communication or crosstalk exists between the neuroimmune and neuroendocrine systems and that the neuroendocrine-immune network plays a vital role in the systems biology of depression [8,9].

Preclinical studies have revealed that hyperactivity of the HPA axis can lead to the activation of the neuroinflammatory response of the immune system, whereas neuroinflammation can also modulate the activity of the HPA axis through various underlying mechanisms [8]. These findings have provided many novel pharmacological targets in either the neuroimmune or neuroendocrine system for depression treatment; however, none of these attempts have succeed in developing new medications directed toward these systems. Because of the intersystem crosstalk, agents that target one system alone will not be effective, and an additional medication that directly acts on the other system is also required to achieve a better treatment. Therefore, an improved approach to achieve an effective depression treatment should be systems biology-orientated and simultaneously target several biological systems involved in the pathogenesis of depression. 

Traditional Chinese Medicine (TCM) is a holistic medicine that has been developed in China for centuries. It emphasizes the integration of a variety of biological systems in the human body and aims to prevent or heal diseases by maintaining or restoring internal homeostasis [4]. In TCM practice, a combination of multiple herbal drugs, so-called Chinese Herbal Medicine (CHM), is often used to act on multiple pharmacological targets simultaneously [10,11]. The systems biology-based, multi-target, and multi-drug medication is particularly suitable for the treatment of multigenetic and multifactorial diseases, such as depression [12].

Numerous CHM formulas are currently used for depression treatment in TCM practice [11,13]. Clinical studies have shown that these CHM antidepressant formulas exert comparable efficacies to conventional antidepressants, but with few adverse effects [14]. In addition, preclinical studies have demonstrated that CHM antidepressant formulas exhibit antidepressant-like activities in rodent models through multiple underlying mechanisms, and the de-hyperactivation of the HPA axis and anti-inflammation are the most common actions [15,16,17]. During the past decade, preclinical studies have extensively been performed by employing the molecular or systems pharmacology approach to uncover the mechanisms of action of CHM antidepressant formulas at both the molecular and systems levels. These studies have not only remarkably improved our understanding of the molecular basis and system-wide actions of CHM antidepressant formulas, but also promoted the development of novel medications for the effective and systematic treatment of depression [4].

In this narrative review, we aim to conclusively uncover the mechanism of action of CHM, specifically through the modulation of the neuroendocrine-immune network, by discussing the recent preclinical studies conducted using depressive animal models. The most recent literature showing that CHM constituents or formulas exert antidepressant activity by modulating the neuroinflammatory response of the immune system or the release of HPA axis hormones were prioritized in the selection of studies for discussion. According to their mechanisms of action, some representative CHM antidepressant constituents and formulas are summarized in each section, respectively. In addition, we also discuss the effects of CHM constituents and formulas on the neuroendocrine-immune network and the systems pharmacology approach in order to improve our understanding of the system-wide mechanisms of action of CHM formulas.

## 2. Inflammation in the Pathogenesis of Depression

Smith first proposed that inflammation may play a crucial role in the pathogenesis of depression in 1991 [18]. Since then, the immune system has been extensively studied to explore the mechanism by which the dysfunction of immune system is associated with symptoms of depression. Accumulating evidence has demonstrated that the dysregulation of the peripheral or neuroimmune system contributes to the pathogenesis of depression [19] (Figure 1). Clinical studies have indicated that patients suffering from depression showed significantly higher levels of proinflammatory cytokines, including interleukin-1β (IL-1β), interleukin-6 (IL-6), tumor necrosis factor alpha (TNF-α), C-reactive protein (CRP), and inflammasome, than healthy people [6,20,21,22,23,24]. These studies have also shown that patients with chronic peripheral inflammatory diseases have a higher incidence of depression [25]. In addition, over 50% of patients suffering from viral infections showed a depressive symptomatology after treatment with cytokine interferon-alpha (INF-α) [26]. The reciprocal effects were also observed in rodent models. For instance, several studies have reported that chronic unpredictable mild stress (CUMS) treatment elevated the proinflammatory or neuroinflammatory response of the immune system in the blood and brain [27,28,29], whereas the administration of endotoxins, such as lipopolysaccharide (LPS), caused depressive-like behaviors by activating the indoleamine 2,3-dioxygenase (IDO) pathway [30] or proinflammatory cytokines [31]. These studies indicate that bidirectional communication exists between proinflammation or neuroinflammation and the CNS.

It is worth noting that the dysregulation of the peripheral immune system plays an important role in the pathogenesis of depression. Peripheral cytokines can be actively transported into the CNS through an increase in blood brain barrier (BBB) permeability [19,32,33] and, subsequently, a reduction of serotonin neurotransmission and activation of the HPA axis [34]. Interestingly, low levels of proinflammatory cytokines regulate PI3K-Akt signaling to support synaptic function; however, abnormally increased proinflammatory cytokines contribute to damage, atrophy, and loss of spinal synapses through the modulation of signaling factors p38 and nuclear factor kappa B (NF-κB) [35].

Preclinical and clinical studies have also demonstrated that stress and depression are associated with an alteration in the morphology and activation of microglial cells, which leads to neuroinflammation and neuronal dysfunction [19,36]. In post-mortem histological and neuroimaging studies on depressive patients, robust changes in the microglial morphology and density in the prefrontal cortex (PFC) and hippocampus have been found [19,37,38]. In addition, a peritoneum injection of LPS into chronic neurodegenerative mice has been shown to result in a dramatic change in the microglia phenotype, which can transform into a proinflammatory phenotype through the overexpression of proinflammatory cytokines, such as IL-1β, IL-6, and TNF-α [39,40]. Furthermore, the activation of microglia mediates depressive-like behaviors through the shaping of the dendritic architecture and synaptic connection [41,42]. Taken together, these findings have provided evidence that the dysfunction of the neuroimmune system is involved in the pathogenesis of depression. 

## 3. CHM Regulation of the Neuroimmune System

**Figure 2:**
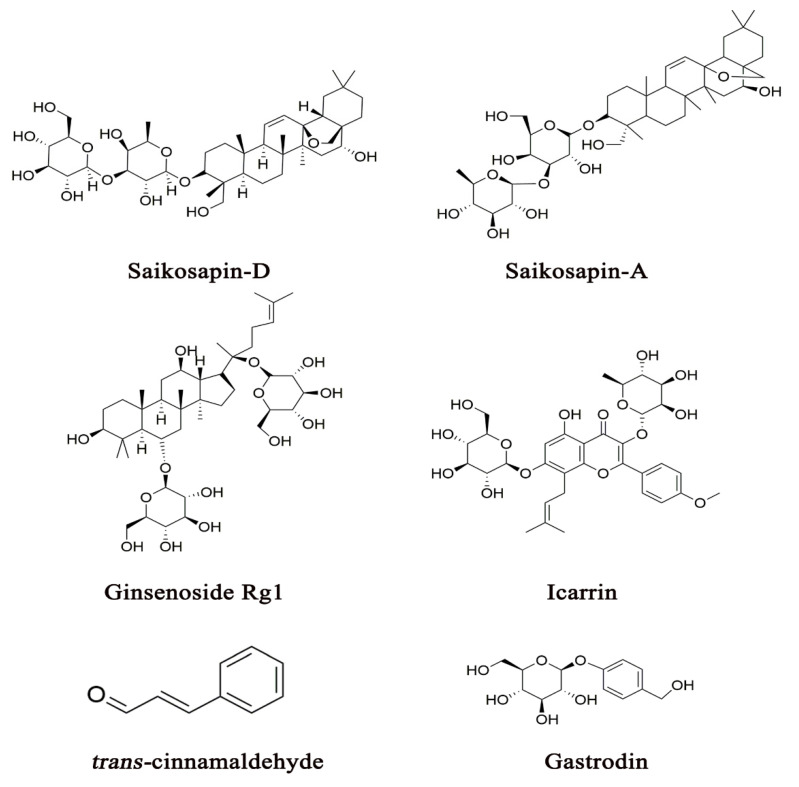
Several representative CHM constituents that have been demonstrated to exert antidepressant activity, specifically by mediating the neuroimmune system.

Many CHM herbal constituents exert anti-inflammation activity through various underlying mechanisms of action by regulating either proinflammatory cytokines, inflammatory signaling pathways or inflammasome [13,43,44,45,46]. While these studies have not revealed the specific drug-target interactions of these herbal constituents with their acting proteins, they have provided the molecular basis for understanding the mechanisms of action by which CHM herbs or formulas exert antidepressant activity through the modulation of the neuroimmune system. Figure 2 shows several representative CHM constituents that have been reported to exhibit antidepressant-like activity in depressive animal models, specifically by mediating the neuroimmune system. Other herbal constituents that possess anti-inflammation activity are also shown in Table 1. Some CHM formulas that produce antidepressant effects through the modulation of the neuroimmune system are listed in Table 2. In this section, we discuss the effects of CHM on the neuroimmune system according to its diverse pharmacological actions.

### 3.1. Proinflammatory Cytokines and Cytokine Receptors

Proinflammatory cytokines are mainly derived from immune cells such as monocytes, macrophages, lymphocytes, and dendritic cells, acting as important modulators of neuroinflammatory response [6]. Recently, several studies have investigated the influence of peripheral proinflammatory cytokines (e.g., IL-6, IL-1β, and TNF-α) on neuronal synaptic plasticity, neurogenesis, and neuromodulation, which play critical roles in the initiation, relapse, and progression of depression [47,48]. It is worth noting that these proinflammatory cytokines are usually maintained at low levels under physiological conditions; however, its levels are increased by approximately 100-fold under stress-related pathological conditions [6]. In addition, microglia can release proinflammatory cytokines that influence the neurobiology of depression by decreasing the number and function of astrocytes [19]. Furthermore, cytokine receptors have also been demonstrated to produce neurobiological effects on microglia activation and neuroinflammation [19]. 

*Bupleurum chinense* DC has commonly been used to treat inflammation and infectious diseases [49]. Saikosaponin-D, a triterpenoid saponin isolated from *Bupleurum chinense* DC, has multiple pharmacological effects, such as anti-inflammation [50] and antidepressant activity [51]. A study has shown that pretreatment with saikosaponin-D (1 mg/kg, 7 days, i.g.) inhibited LPS-induced microglia activation and suppressed the secretion of proinflammatory cytokines (IL-1β, IL-6, and TNF-α) [52]. Its mechanism of action has been suggested to inhibit the transportation of high mobility group box 1 (HMGB1), a late inflammatory factor, into extracellular space, which results in the downregulation of the Toll-like receptors 4 (TLR4)/NF-κB pathway in both the hippocampus in mice and primary microglia cells. Saikosaponin-A (50 or 100 mg/kg, 4 weeks, p.o.), a derivative of saikosaponin-D, has also been demonstrated to exhibit antidepressant-like activity through its effects on the neuroimmune system by suppressing the CUMS-induced IL-1β, IL-6, and TNF-α overexpression in the hippocampus in rats [53]. Taken together, these results indicate that triterpenoid saponins exert antidepressant-like activities in animal models by reducing proinflammatory cytokine levels; however, further study is needed to explore their molecular interactions with the potential acting proteins and their pharmacological profiles.

While no CHM constituents have been reported to exert a specific action on cytokine receptors, a CHM formula comprised of eight herbs for the treatment of postpartum depression, known as the Shen-Qi-Jie-Yu decoction (1.25 g/mL, 1, 2, 4 weeks, i.g.), has recently been shown to produce antidepressant effects by decreasing the expression of cytokine receptors, such as IL-1R_1_ and glycoprotein 130, in the hippocampus in a rat model of postpartum depression [54]. However, this study did not clarify the molecular mechanism by which the herbal formula produces profound effects on the expression of cytokine receptors. 

### 3.2. Proinflammatory Signaling Pathway

Increasing evidence has suggested that proinflammatory signaling pathways, such as the mitogen-activated protein kinase (MAPK) pathway and NF-κB pathway, influence BBB integration, microglia activation, and neurogenesis [6,55]. A bioinformatic analysis has identified that the MAPK pathway is one of the functionally enriched signaling pathways in the neurobiology of depression [56]. Extracellular regulated kinases (ERK1/2), c-Jun N-terminal kinases (JNKs), and p38 MAP kinases are three subfamilies of the MAPK signaling pathway. It has been demonstrated that the MAPK pathway is involved in the differentiation of astrocytes and other neuronal cells, synaptic plasticity, and neuron survival, as evidenced by the robust changes of MAPK signaling in the hippocampus in depressive animal models [6,57,58,59]. These kinase subfamilies play central roles in the release of proinflammatory cytokines or the activation of NF-κB, a proinflammatory transcription factor, in response to various stimuli, such as psycho-emotional stressors, acute alcohol exposure, pathogenic products, or proinflammatory cytokines [60,61,62]. NF-κB is abundantly distributed in the brain, microglia, BBB, and peripheral immune responsive cells [60,61,63], and it is an essential mediator in several important physiological processes, including synaptogenesis, neurotransmission, neuroprotection, and neuroinflammation [64,65,66]. In animal models, NF-κB activity can be elevated by the degradation of NF-κB kinase inhibitors [67], and it can also be activated by IL-1β signaling and proinflammatory cytokines released from both the peripheral immune cells and the brain [68,69]. A study showed that proinflammatory cytokine overexpression and behavioral abnormality can be reduced by the blockade of NF-κB signaling [68]. Hence, these findings highlight the critical roles of the MAPK and NF-κB signaling pathways in the pathogenesis of depression.

Many CHM herbal constituents have been shown to exert antidepressant-like activities in animal models by inhibiting the MAPK or NF-κB signaling pathway [70,71]. Ginsenoside Rg1, a saponin extracted from *Panax ginseng* C. A. Mey., has been demonstrated to act as a potential neuroprotective agent in depression treatment [72,73]. The administration of ginsenoside Rg1 (20 or 40 mg/kg, 34 days, i.g.) inhibited hippocampal neuroinflammation and reduced the expression of proinflammatory cytokines and microglial activation through the inhibition of NF-κB activity by the MAPK and silent information regulator 2 homolog 1 signaling pathways in chronic social defeat stress mice [72]. Additionally, ginsenoside Rg1 (10 or 20 mg/kg, 3 days, p.o.) has also been revealed to significantly reduce the p-IκB level in cytoplasm and nuclear translocation of NF-κB in LPS-induced depressive mice, probably through the suppression of iNOS and TNF-α production in the brain [74]. Moreover, in comparison with the LPS-induced model, the levels of the MAPK subfamily, such as p38 MAPK, ERK, and JNK, were reversed through treatment with Rg1 [74]. These studies have indicated that the inhibitory effects of Rg1 are meditated by inhibiting the NF-κB and MAPK pathways. While these findings have provided novel insights into the therapeutic implications of ginsenoside Rg1 for depression treatment, further study is needed to determine if the herbal constituent directly interacts with the neuroinflammatory signaling pathways and what pharmacological targets it specifically acts on. 

### 3.3. Inflammasome

Inflammasome, a complex of multiple proteins, functions as an intracellular sensor in response to environmental and cellular stress [75,76,77]. In particular, the NLRP3 inflammasome complex, the well-studied inflammasome member, acts as a key convergent molecular pathway in several mechanisms of peripheral and central inflammatory responses in neurological and inflammatory diseases [78]. NLRP3 inflammasome can be released by microglia, macrophages, and astrocytes in the CNS [77]. NLRP3 activation, induced by the stimulation of either Toll-like receptors (TLRs) or adenosine triphosphate (ATP), results in caspase-1 activation and IL-1β maturation, which initiate inflammatory responses [77]. Clinical studies have demonstrated that the gene expression of NLRP3, caspase-1, and IL-1β are elevated in blood samples of depressive patients [79]. In addition, it has also been shown that, in rodent models, the NLRP3 inflammasome is significantly elevated in depressive brain tissues and that the administration of an NLRP3 inhibitor improves depressive-like behaviors induced by LPS or CUMS [80,81]. Furthermore, Su et al. has suggested that NLRP3 inflammasome modulates depressive-like behaviors through the regulating activities of the MAPK and NF-κB pathways [55]. Taken together, these studies indicate that the activation of NLRP3 inflammasome signaling is involved in the pathogenesis of depression and that inflammasome serves as a potential pharmacological target in depression treatment.

Studies have shed light on depression treatments involving CHM constituents that suppress the overexpression or activation of NLRP3. For example, *trans*-cinnamaldehyde (10 μM, 24 h), a bioactive constituent of *Cinnamomum cassia* Presl, has been shown to inhibit microglia activation and, subsequently, to alleviate inflammatory responses to various stressors [82]. The administration of *trans*-cinnamaldehyde (10 mg/kg, 3 weeks, p.o.) has been shown to increase sucrose preference and reduce the immobility time in CUMS rats. The study has also shown that *trans*-cinnamaldehyde attenuated the expression of NF-κB pathway components, including TLR-4, IκBα, p65, NF-κB-1, and TNF-α, and downregulated the expression of NLRP3, caspase-1, IL-1β, and IL-18. These observations suggest that *trans*-cinnamaldehyde produces antidepressant effects through the inactivation of the NF-κB/NLRP3 inflammasome pathway in animal models [28]. Moreover, a recent study has suggested that icariin (20 or 40 mg/kg, 35 days, p.o.), a prenylated flavonoid extracted from *Epimedium brevicornu* Maxim, exerts anti-inflammation effects and ameliorates oxidative stress-induced brain damage by inactivating the NF-κB signaling and inhibiting the NLRP3-inflammasome/caspase-1/IL-1β axis in the hippocampus [83]. It would be interesting to identify their specific targeting proteins in the inflammasome complex, which would, in turn, improve our understanding of the mechanisms of action by which CHM constituents modulate the neuroimmune system by specifically interacting with inflammasomes.

**Table 1:** Constituents of CHM that modulate the release of HPA axis hormones and exhibit anti-inflammatory and antidepressant-like activities in animal models of depression.

Herb	Herbal Constituent	Animal Model	Behavioral Test	Administration Dose/Time/Route of Treatment	Effects on Mediators of Inflammation	Effects on Hormones of the HPA Axis	Reference
*Fallopia multiflora* (Thunb.) Harald.	2, 3, 5, 4′-Tetrahydroxystilbene-2-O-β-D-glucoside	LPS-induced depressive mice	TPT, FST, SPT	30, 60 mg/kg, 7 days, i.p.	↓ IL-6, TNF-α, IL-1β in hippocampus and PFC	ND	[84]
*Acanthopanax sessiliflorus* (Rupr. et Maxim.) Seem.	Chiisanoside	LPS-induced depressive mice	TST, FST	2.5, 5.0 mg/kg, 7 days, i.p.	↓ IL-6, TNF-α in serum ↓ NF-κB in hippocampus	ND	[85]
*Polygala tenuifolia* Willd.	Senegenin	CUMS mice	TST, FST, SPT	4, 8 mg/kg, 21 days, i.g.	↓ NF-κB/NLRP3 signal pathway in hippocampus	ND	[86]
*Gastrodia elata* Bl.	Gastrodin	CUS rats	SPT, FST, Morris water test	50, 100, 200 mg/kg, 14 days, i.p.	↓ NF-κB and IL-1β expression in hippocampus	ND	[87]
*Cinnamomum cassia* Presl	Trans-Cinnamaldehyde	CUMS rats	Sucrose consumptions, FST	10 mg/kg, 3 weeks, p.o.	↓ IL-1β, IL-18, TNF-α in serum ↓ NF-κB/NLRP3 in PFC and hippocampus	ND	[28]
*Lonicera japonica* Thunb.	Lonicera japonicapolysaccharide	CUMS mice	OFT, EPM, TST, FST	30, 100 mg/kg, 21 days, i.g.	↓ NLRP3, IL-1β, caspase-1 in hippocampus	ND	[88]
*Andrographis paniculata* (Burm. f.) Nees	Andrographolide	CUMS mice	FST, SPT, TST, Y maze	2.5, 5 mg/kg, 14 days, p.o.	↓ IL-1β, IL-6, TNF-α, NF-κB signaling, NLRP3 in PFC	ND	[89]
*Houpoea officinalis* (Rehder and E. H. Wilson) N. H. Xia and C. Y. Wu	Honokiol	LPS-induced depressive mice	FST, TST,	2.5, 5 10 mg/kg, 11 days, p.o.	↓ NF-κB activation in hippocampus ↓ IL-1β, TNF-α, IFN-γ in serum	ND	[90]
*Gardenia jasminoides* Ellis /*Crocus sativus* L.	Crocin	LPS-induced depressive mice	SPT, FST, TST, OFT	20, 40 mg/kg, 7 days, i.p.	↓ TNF-α, IL-1β, IL-18 in BV-2 microglial cells and hippocampus ↓ NF-κB and NLRP3 in hippocampus	ND	[91]
*Perilla frutescens* (Linn.) Britt.	Perilla aldehyde	CUMS rats	SPT, FST, OFT	20, 40 mg/kg, 3 weeks, i.g.	↓ TNF-α, IL-1β in hippocampus ↓ NLRP3 in hippocampus	ND	[92]
		LPS-induced depressive mice	TST, FST	60, 120 mg/kg, 7 days, i.g.	↓ TNF-α, IL-6 in serum and PFC	ND	[93]
	Essential oil of *Perilla frutescens*	CUMS mice	OFT, TST, FST, SPT	3, 6, 9 mg/kg, 3 weeks, g.i.	↓ TNF-α, IL-6, IL-1 in plasma	ND	[94]
*Polygonum aviculare* L.	*Polygonum aviculare* L. extract	Restraint-stressed mice	FST, SPT, OFT	100, 200 mg/kg, 15 days, p.o.	↓ TNF-α, IL-6, IL-1β in the brain	ND	[95]
*Hemerocallis fulva* (L.) L.	Ethanol extracts	LPS-induced depressive mice	SPT	180 mg/kg, 7 days, p.o.	↓ NF-κB signaling pathway in PFC	ND	[96]
*Angelica sinensis* (Oliv.) Diels	Ferulic Acid	CUMS mice	SPT	20, 40, 80 mg/kg, 4 weeks, p.o.	↓ TNF-α, IL-6, IL-1β, microglial activation, NF-κB and NLRP3 in PFC	ND	[97]
*Paeonialactiflora* Pall	Paeoniflorin	IFN-α-induced depressive mice	SPT, OFT, TST, FST	10, 20, 40 mg/kg, 4 weeks, i.g.	↓ TNF-α, IL-6, IL-1β, IL-9, IL-10, IL-12, MCP-1 in serum, mPFC, vHi and amygdala	ND	[98]
Xiaobuxin-Tang	Total flavonoid extract	LPS-induced depressive mice	TST, FST	25, 50, 100 mg/kg, 1 h, i.p.	↓ TNF-α, IL-1β in the barin	ND	[99]
*Ginkgo biloba* L.	EGb761	LPS-induced depressive mice	FST, TST, SPT	50, 100, 150 mg/kg, 10 days, p.o.	↓ IL-6, TNF-α, IL-1β, IL-17A in hippocampus ↑ IL-10 in hippocampus	ND	[100]
*Pueraria lobate* (Willd.) Ohwi	Puerarin	CUS rats	SPT, NSFT, FST	30, 60, 120 mg/kg, 20 days, i.g.	ND	↓ CRH, CORT, ACTH in serum	[101]
*Tribulus terrestris* Linnaeus	Tribulus Terrestris Saponins	CMS rats	OFT, SPT	0.375, 0.75, 2.25 g/kg, 4 weeks, i.g.	ND	↓ CRH, CORT in serum	[102]
*Rehmannia glutinosa* (Gaert.) Libosch. ex Fisch. et Mey.	Ethanol extracts	CUMS rats	SPT	150, 300, 600 mg/kg, 3 weeks, p.o.	ND	↓ CORT in serum	[103]
*Panax ginseng* C.A. Meyer	Ginseng total saponins	LPS-induced depressive mice/RAW264.7 cells; CUMS rats	FST, TST, SPT	200 mg/kg, 7 days, i.g.; 12.5, 25, 50 mg/kg, 6 weeks, i.g.	↓ IL-1β, IL-6, TNF-α, IDO mRNA in hippocampus	↓ CORT in serum ↑ GR mRNA in hippocampus	[104,105]
	Ginsenoside Rg1	CSDS mice; CUMS rats	Social interaction test, SPT, FST, TST	20, 40 mg/kg, 34 days, i.g.; 5, 10, 20 mg/kg, 28 days, i.g.	↓ IL-6, TNF-α, IL-1β, microglial activation, p-NF-κB in hippocampus	↓ CORT level in serum ↑ GR protein in PFC and hippocampus	[72,106]
	Ginsenoside Rg3	LPS-induced depressive mice; CUS rats	TPT, FST, EPMT, NSFT, OFT	20, 40 mg/kg, 3 days, i.g.; 10, 20, 40 mg/kg, 14 days, i.g.	↓ IL-6, TNF-α in plasma ↓ IL-6, IL-1β, IDO, microglial activation, NF-κB pathway in brain	↓ CRH, CORT, ACTH in serum	[107,108]
*Salvia miltiorrhiza* Bunge	Salvianolic acid B	CMS mice	SPT, FST, TST	20 mg/kg, 3 weeks, i.p.	↓ IL-1β, TNF-α in hippocampus and cortex ↑ IL-10, TGF-β in hippocampus and cortex	↓ CORT in plasma	[109]
*Aquilaria* spp.	Agarwood Essential Oil	Restraint stress-induced mice	TST, FST	10, 20, 40 mg/kg, 10 days, i.p.	↓ IL-1β, IL-1α, IL-6 in serum	↓ CRF, CRF receptor in cortex ↓ CORT, ACTH in serum	[110]
Chaihu-Shugan-San	Saikosaponin A	CUMS rats	SPT, NPFT, FST	25, 50 or 100 mg/kg, 4 weeks, p.o.	↓ IL-1β, IL-6, TNF-α in hippocampus	↓ CRH in hypothalamus ↓ GR mRNA in hippocampus	[53]
*Rhodiola rosea* L.	Salidroside	OBX rats	TST, FST, SPT	20, 40 mg/kg, 2 weeks, p.o.; 20, 40 mg/kg, 2 weeks, i.g.	↓ TNF-α, IL-1β in hippocampus ↓ IL-1β, IL-6, TNF-α, NF-κB activation in PFC	↑ GR in hippocampus ↓ CRH in hypothalamus	[111,112]
*Epimedium brevicornu* Maxim.	Icariin	CMS rats; CSD mice	SPT, FST, social avoidance evaluations	20, 40 mg/kg, 35 days, p.o.; 25, 50 mg/kg, 28 days, i.g.	↓ IL-1β, TNF-α, NF-κB signaling pathway, NLRP3/caspase-1/IL-1β axis activation in hippocampus	↓ CORT, IL-6 in serum ↑ GR in livers	[83,113]
*Curcuma longa* L.	Curcumin	CUMS rats; CUS rats	SPT, FST, EPM, Shuttle-box testing	40 mg/kg, 5 weeks, i.p.; 100 mg/kg, 4 weeks, i.g.; 2.5, 5, 10 mg/kg, 21 days, p.o.	↓ TNF-α, IL-1β, IL-6, NF-κB in mPFC ↓ TNF-α, IL-1β, IL-6 mRNA, NLRP3 in hippocampus	↓ CORT in serum ↑ GR mRNA in serum	[114,115,116]
*Polygonum cuspidatum* Siebold et Zucc.	Resveratrol	Ouabain-induced depressive mice; Hippocampal neuron cells; CUMS rats	OFT, EPM, Barnes maze performance, object recognition, passive avoidance experiments, SPT, FST	10 mg/kg, 10 weeks, p.o.; 15 mg/kg, 21 days, i.g.	↓ IL-1β, IL-17A, IL-8, TNF-α in serum and hippocampal neuron cells	↓ CORT in serum ↓ CRF mRNA in hypothalamus	[117,118]
*Bupleurum chinense DC.*	Saikosaponin D	LPS-induced depressive mice; UCMS rats	SPT, TST, FST, OFT	1 mg/kg, 7 days, i.g.; 0.75, 1.5 mg/kg, 21 days, i.g.	↓ microglia activation in hippocampus ↓ IL-6, TNF-α, IL-1β in vivo and vitro ↓ TLR4/NF-κB signaling pathway in hippocampus	↓ CORT in serum ↑ GR in hippocampus	[52,119]
*Scutellaria baicalensis* Georgi	Baicalin	CUMS mice; CUMS rats; CORT-induced depressive-like mice	SPT, OFT, TST FST	60 mg/kg, 14 days, i.g.; 20, 40 mg/kg, i.g., 3 weeks; 10, 20 mg/kg, 21 days, i.g.	↓ IL-1β, TNF-α, IL-6, TLR4 in the hippocampus ↓ GSK3β/NF-κB/NLRP3 signal pathway in hippocampus	↓ GR mRNA, GRα in hippocampus	[120,121,122]

**Table 2:** CHM formulas traditionally used in TCM for the treatment of depression, which exhibit anti-inflammatory activity and modulate the release of HPA axis hormones.

CHM Formula	Plant Name/Ratio in Fixed Combination	Daily Human Dose	Animal Model	Behavioral Test	Administration Dose/Time/Route of Treatment	Effects on Mediators of Inflammation	Effects on Hormones of the HPA Axis	Reference
Xiaoyao Pills	*Bupleurum chinense* DC., *Osmanthus fragrans* var. *aurantiacus* Makino, *Paeonia lactiflora* Pall., *Smilax glabra* Roxb, *Atractylodes macrocephala* Koidz., *Mentha haplocalyx* Briq., *Zingiber officinale* Roscoe and *Glycyrrhiza uralensis* Fisch.; 3:3:3:3:3:1:2:1.5	2 times/day	LPS-induced depressive mice/rats	TST, FST, OFT, NSFT	0.4836, 0.93, 1.86 g/kg, 14 days, i.g.	↓ IL-6 in serum and hippocampus ↓ TNF-α in hippocampus and cortex	ND	[123,124,125]
Mahuang-Fuzi-Xixin Decoction	*Aconitum carmichaeli* Pcbx., *Ephedra sinica* Stapf and *Asarum sieboldii* Miq.; 3:2:1	3 times/day	LPS-induced depressive mice	SPT, OFT, TST, FST	2.5, 12.5, 25 g/kg, 1 week, p.o.	↓ IL-1β, NLRP3 in hippocampus	ND	[126]
Jieyu Anshen granule	*Bupleurum abchasicum* Manden., *Ziziphus jujuba* Mill., *Dens* Draconis, *Polygala tenuifolia* Willd., *Lilium brownie* var. *viridulum* Baker, *Atractylodes macrocephala* Koidz., *Triticum aestivum* L., *Angelica sinensis* (Oliv.) Diels, Acorus tatarinowii Schott, *Pinellia ternate* (Thunb.) Makino, *Glycyrrhiza uralensis* Fisch., *Gardenia jasminoides* J. Ellis, *Arisaema Cum* Bile, *Curcuma longa* L., *Smilax glabra* Roxb., *and Fructus* Jujubae.; 4:5:10:4:10:3:10:3:4:3:3:4:4:4:5:3	5 g; 2 times/day	PSD rats	OFT, SPT, water maze test	1, 3 g/kg, 4 weeks, i.g.	↓ NF-κB signaling in PFC and hippocampus	ND	[127,128]
Jiaotai wan	*Coptis chinensis* Franch. and *Cinnamomum* cassia.; 10:1	1.5–2.5 g/day	LPS-induced depressive mice	TST, FST, SPT, OFT	4.2, 8.4 g/kg, 7 days, i.g.	↓ TNF-α, IL-6 in serum ↓ NF-κB signaling in brain	ND	[129,130]
Shen-Qi-Jie-Yu Decoction	*Astragalus membranaceus* (Fisch) Bunge, *Curcuma aromatica* Salisb, *Ziziphus jujuba* var spinosa (Bunge) Hu ex HF Chow, *Cornus officinalis* Sieb et Zucc (Cornaceae), *Codonopsis pilosula* (Franch) Nannf, *Citrus reticulata* Blanco, *Citrus medica* L, and *Angelica sinensis* (Oliv) Diels.; 10:7.5:7.5:7.5:6:5:5:5	1 time/day	Postpartum depressive rat model	OFT, SPT, FST	1.25 g/mL, 1, 2, 4 weeks, i.g.	↓ IL-1β and IL-6 in serum ↓ IL-1RI and gp130 in hippocampus	ND	[54]
Jieyuanshen Decoction	*Bupleurum chinense* DC., *Scutellaria baicalensis* Georgi, *Ziziphusjujuba* Mill. var. spinosa (Bunge) Hu ex H.F. Chou, *Glycyrrhiza uralensis* Fisch., *Lilium brownie* F.E. Brown var. viridulum Baker, and *Pinelliaternata* (Thunb.) Breit.; 1:1.5:0.5:1:1:3	2 times/day	CUS rats	SPT, OFT	8.2, 16.3, 32.7 g/kg, 28 days, i.g.	ND	↓ CORT, ACTH, CRH in serum ↑ GR in hippocampus	[131]
Zhizihoupo Decoction	*Gardenia jasminoides* Ellis, *Citrus aurantium* L., and *Magnolia officinalis* Rehd. et Wils.; 1:1:7	2 times/day	CUMS rats	SPT, FST, OPT	3.66, 7.32, 14.64 g/kg, 3 weeks, i.g.	ND	↓ ACTH, CORT in plasma	[132]
Shuyu San	*Bupleurum chinense* DC., *Curcuma aromatica* Salisb., *Mentha canadensis* Linnaeus, *Gardenia jasminoides* Ellis, *Smilax glabra* Roxb., *Polygala tenuifolia* Willd., *Acorus gramineus* Soland., *Ziziphus jujuba* var. spinosa (Bunge) Hu ex H. F. Chow., and *Albizia julibrissin* Durazz.; 5:7.5:3:5:5:5:5:7.5:5	2 times/day	UCMS rats	TST, FST	2.5, 7.5, 25 g/kg, 3 weeks, g.p.	ND	↓ CRH, ACTH, CORT in serum	[133]
Chaihu-Shugan-San	*Bupleurum chinense* DC., *Citrus reticulata* Blanco, *Ligusticum sinense* ‘Chuanxiong’, *Cyperus rotundus* L., *Citrus × aurantium* Linnaeus, *Paeonia lactiflora* Pall., and *Glycyrrhiza uralensis* Fisch.; 4:4:3:3:3:3:1	2 times/day	ApoE^-/-^ mice; UMS rats	SPT, OFT, LDET, TST	3, 9 g/kg, 16 weeks, i.g.; 5.9 g/kg, 2 weeks, i.g.	↓ TNF-α, IL-1β, IL-6 in plasma and hippocampus	↓ CRH, ACTH in plasma	[124,134,135,136,137]
Kaixin San	*Panax ginseng* C.A. Meyer, *Poria cocos* (Schw.) Wolf, *Polygala tenuifolia* Willd, and *Acorus tatarinowii* Schott.; 1:1:25:50 or 3:2:2:3 or 1:1:1:2	2 times/day	CUMS rats; CUMS rats	SPT	338, 676 mg/kg, 3 weeks, p.o.; 3, 10 g/kg, 6 weeks, i.g.	↓ COX-2, IL-2, IL-6, TNF-α in serum and hippocampus ↑ IL-10, IFN-γ in hippocampus and serum	↓ CRH, ACTH, CORT in serum and organs	[29,138,139,140]
Si-Ni San	*Citrus aurantium* L., *Bupleurum chinense* DC., *Paeonia lactiflora* Pall., and *Glycyrrhiza uralensis* Fisch.; 2:2:3:2	2 times/day	Reserpine-induced rats; Mice	FST, SPT, OFT, TST	0.75, 1.5, 3.0 g/kg, 2 weeks, p.o.; 325, 650, 1300 mg/kg, 60 min, p.o.	↓ IL-1β, IL-6, TNF-α in serum, liver, and hippocampus ↓ NF-κB in hippocampus	↓ CORT in serum	[16,17,141]
Banxia houpo Decoction	*Pinellia ternate* (Thunb.) Breit., *Smilax glabra* Roxb, *Houpoea officinalis* (Rehder and E. H. Wilson) N. H. Xia and C. Y. Wu, *Zingiber officinale* Roscoe, and *Folium* Perillae; 4:4:3:3:2	2 times/day	CUMS rats	SPT	3.29, 6.58 g/kg, 6 weeks, i.g.	↓ NLRP3 activation in livers, hypothalamus, PFC	↓ CORT, CRF in serum	[15,142]

Notes: The conversion ratio of CHM formulas between human and animals should be calculated according to the following formulas: human dose (mg/kg) to mice dose (mg/kg): multiply by 12.3; human dose (mg/kg) to rat dose (mg/kg): multiply by 6.2 [143]. ↓ means decrease; ↑ means increase. Abbreviation in tables: CUMS, chronic unpredictable mild stress; UCMS, unpredictable chronic mild stress; CSD, chronic social defeat; CSDS, chronic social defeat stress; CMS, chronic mild stress; SDM, social defeat model; CUS, chronic unpredictable stress; PSD, poststroke depression; LPS, lipopolysaccharides; OBX, olfactory bulbectomized; CORT, corticosterone; GR, glucocorticoid receptor; ACTH, adrenocorticotropin; CRF, corticotrophin releasing factor; CRH, corticotrophin releasing hormone; SPT, sucrose preference test; TST, tail suspension test; OFT, open field test; FST, forced swimming test; EMP, elevated plus maze; NSFT, novelty suppressed feeding test; NIHT, novelty induced hypophagia test; LDET, light dark exploration test; IL, interleukin; TNF-α, tumor necrosis factor-α; IFN-γ, Interferon gamma; NLRP3, NOD-like receptor protein 3; COX-2, cyclooxygenase-2; NF-κB, nuclear factor kappa B; TLR4, toll-like receptor 4; IDO, indoleamine 2,3-dioxygenase; GSK3β, Glycogen synthase kinase-3β; PFC, prefrontal cortex; vHi, ventral hippocampus; ND, not determined; i.p., intraperitoneal; i.g., intragastrically; p.o., peros; g.p., gastric perfusion; g.i., gastric intubation.

## 4. CHM Modulation of the HPA Axis

It is well known that hyperactivation of the HPA axis in the neuroendocrine system, induced by acute or chronic stress, is a common feature in depressive patients. In response to these somatic stimuli, corticotrophin releasing factor (CRF) or corticotrophin releasing hormone (CRH) is secreted from the median paraventricular nucleus in the hypothalamus and then activates the pituitary to synthesize and release adrenocorticotropic hormone (ACTH). ACTH further activates the adrenal cortex to release glucocorticoid (corticosterone (CORT) or cortisol), which, in turn, regulates the HPA axis through a negative feedback loop at multiple levels: directly on elements of the axis and indirectly through the PFC, amygdala, and hippocampus [144,145,146] (Figure 1). Studies have demonstrated that hyperactivity of the HPA axis reduces synaptic function, atrophies neurons, and subsequently results in depressive behaviors [147]. Additionally, excessive CRF secretion induced by the desensitization of CRF pituitary receptors, leads to high concentrations of CRF in the CNS, which contributes to the risk of depression [148,149]. Moreover, the abnormal activation of the HPA axis can also be induced by the downregulation of glucocorticoid receptors (GR) in the hippocampus [150]. These studies have provided several pharmacological targets to suppress stress-induced hyperactivation of the HPA axis, but attempts to develop novel agents directed toward the HPA axis in the treatment of depression have not been successful.

**Figure 3:**
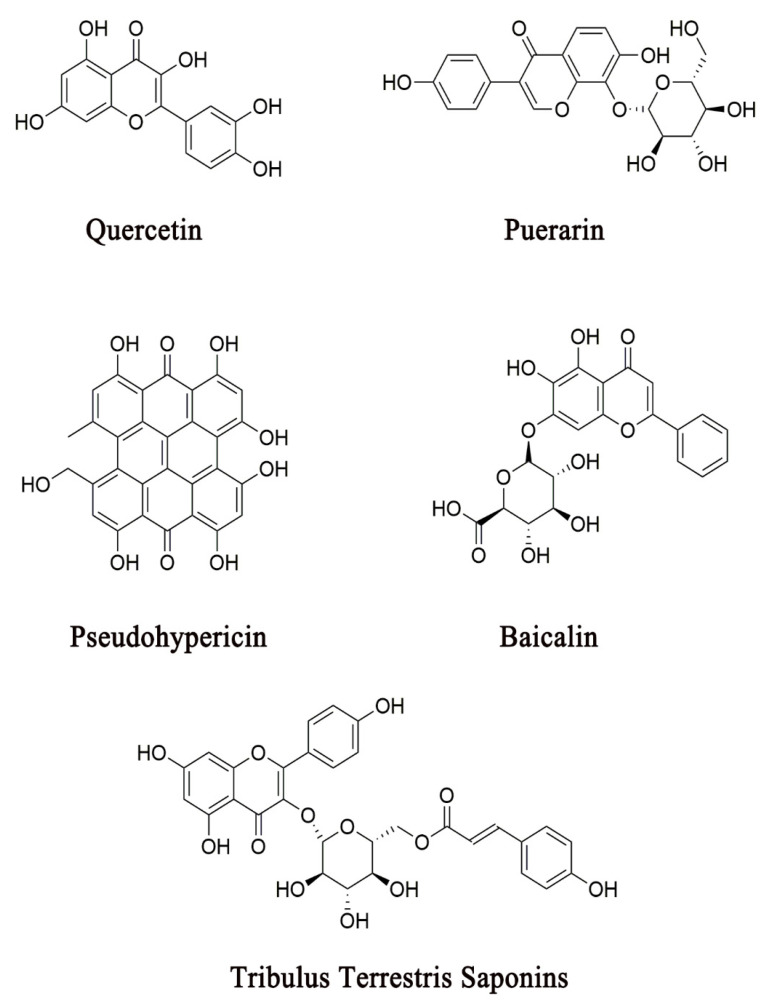
Several representative CHM constituents that have been demonstrated to exhibit antidepressant-like activities through their action on HPA axis hormone receptors.

On the other hand, preclinical studies have demonstrated that many CHM herbal constituents or formulas can attenuate depressive-like symptoms through the modulation of the activity of the HPA axis in depressive animal models [101,102,131,132,133]. These findings have provided the molecular basis for understanding the mechanism of action of CHM in the treatment of depression, by which CHM constituents or formulas produce antidepressant activities by specifically acting on modulators of the HPA axis. In this section, we review the roles that CHM play in the modulation of the activity of the HPA axis. Several representative CHM constituents that have been demonstrated to target the hormone receptors in the HPA axis are shown in Figure 3. Other CHM constituents or formulas that modulate the release of HPA axis hormones are listed in Table 1 and Table 2, respectively. 

### 4.1. CRF Antagonists

CRF is commonly considered to be a vital factor in response to stress at the neural, endocrinological, and immunological levels [9]. It is noticeable that CRF is involved in the structural integrity of the brain and in the regulation of neurotransmitter transmission [151]. Studies have demonstrated that CRF antagonists could be potential antidepressants that alleviate depressive symptoms through the suppression of the hyperactivation of the HPA axis [152].

Quercetin, a flavonoid abundantly distributed in many herbs, has been shown to exhibit anxiolytic- and antidepressant-like activities in animal models by antagonizing the effect of CRF [153]. In this study, the administration of quercetin (20 or 40 mg/kg, 60 min, p.o.) significantly reduced the levels of CORT and adrenocorticotropic hormone in plasma and the mRNA expression of CRF in the hypothalamic region in water immersion-restraint rats. It also demonstrated that quercetin suppressed CRF expression, probably through the modulation of the DNA-binding activity of the glucocorticoid receptor and the phosphorylation of the cyclic adenosine 3′,5′-monophosphate (cAMP) response element-binding protein and extracellular signal-regulated kinase 1/2 in the hypothalamic region [154]. While this study has provided experimental evidence that quercetin acts as a modulator antagonizing the effect of CRF in the HPA axis, the specific drug–target interaction remains to be uncovered.

### 4.2. Corticotrophin Releasing Factor 1 (CRF_1_) Receptor Antagonists

After its release, CRF binds to two major receptors: CRF_1_ and CRF_2_. It is noteworthy that the CRF_1_ receptor is widely distributed in the brain, while the CRF_2_ receptor is highly expressed in peripheral tissues [9,155]. Increasing evidence has suggested that an elevated CRF_1_ receptor function, rather than the CRF_2_ receptor, is involved in the pathogenesis of anxiety and depression [156]. Furthermore, several lines of preclinical evidence has shown that knockout of the CRF_1_ receptor in mice produces anti-anxiety effects [9], while CRF_2_ receptor-deficient mice exhibit increased anxiety- and depressive-like behaviors [9,157,158]. Thus, the CRF_1_ receptor seems to be a key receptor for the HPA axis in the pituitary in response to stress, and the blocking CRF_1_ receptor has been proposed to be an effective therapeutic approach in depression treatment.

In response to stress, CRF initiates the activity of the HPA axis through by binding to the CRF_1_ receptor in the anterior pituitary and thus activating adrenocorticotropic hormone secretion [159]. Clinical studies have suggested that the CRF_1_ receptor plays a crucial role in individuals’ risk of developing depression [151]. It is noteworthy that CRF_1_ receptor antagonists have been tested for their efficacy in depression treatment, but the results were inconsistent. One study has shown that the administration of NBI-30775/R121919 (40–80 mg/day for 30 days), a CRF_1_ receptor antagonist, significantly attenuated depressive symptoms in patients [160]. However, in another study, the authors did not observe the antidepressant effects of CRF_1_ receptor antagonists, such as CP-316311 [161]. It has also been demonstrated that some CRF_1_ receptor antagonists, including antalarmin, CP154,526, and R121919, did not produce antidepressant-like effects in rat models [162]. These studies have argued that treatment with CRF_1_ antagonists is only beneficial for depressive patients with CRF overactivity [160,161].

CHM constituents have been shown to produce antidepressant effects in animal or cell models by antagonizing the CRF_1_ receptor [9,110,163]. The activity of three major constituents isolated from *St. John’s* wort, hypericin, pseudohypericin, and hyperforin against the CRF_1_ receptor has been examined by measuring their effects on CRF-stimulated cAMP formation [163]. This study showed that only pseudohypericin (10 μM) selectively inhibits CRF_1_ receptor activity, but hypericin and hyperforin antagonizes both CRF and calcitonin [163]. To our knowledge, pseudohypericin is the first herbal molecule to be identified as a CRF_1_ receptor antagonist. 

### 4.3. GR Agonists or Antagonists

GR is a glucocorticoid receptor that is distributed in the HPA axis. Hyperactivation of the HPA axis can impair GR function because of the elevated cortisol and glucocorticoids [164]. Additionally, GR dysfunction may also result from decreased glucocorticoid binding to GR or decreased GR expression in the HPA axis [165,166]. It has been observed that, in comparison with healthy controls, GR mRNA levels are decreased in the brain regions of depressed patients in postmortem studies [167,168]. Another study showed that treatment with antidepressants can increase GR binding and GR mRNA expression in the brain, thus ameliorating depressive symptoms [169]. Thus, the upregulation of GR expression and function through, for example, a GR agonist, has been proposed to be pivotal for the therapeutic mechanism of antidepressants. However, a clinical study involving 490 patients with depression indicated that either an increased or decreased GR mRNA results in a greater susceptibility to depression [170]. It is noticeable that polymorphisms of the GR gene play a critical role in the pathogenesis of depression [170]. Hence, GR antagonists have also been recognized to be potential modulators in the development of antidepressants. Clinical studies have shown that a GR antagonist, mifepristone, ameliorates psychotic symptoms and cognitive deficits in patients with depression or bipolar disorders [171,172]. Preclinical studies have also demonstrated that GR deficits in the PFC of mice resulted in depressive-like behaviors [173], which can be ameliorated by the administration of mifepristone [173]. However, in a clinical Phase III study, mifepristone was found to have disappointing effects in terms of the effective reduction of psychotic symptoms in depression sufferers [174], and its abortifacient properties severely compromised its use in women with depression [175]. Thus, mifepristone has not been recognized as an antidepressant drug on the market.

CHM herbal constituents or formulas have been reported to attenuate depressive behaviors through the modulation of stress-impaired GR in animal models. The administration of baicalin (20 mg/kg, 21 days, p.o.), a major constituent in *Scutellaria baicalensis* Georgi, has been demonstrated to significantly attenuate CORT-induced behavioral abnormalities through the upregulation of GR mRNA and GRα expression in the hippocampus in mice [122]. In addition, a CHM formula, known as the Huang-Qin-Hua-Shi decoction (1 mL/100 g, 3 weeks, i.g.), has also been shown to block the high-temperature- and high-humidity-stress-induced upregulation of hypothalamus GR mRNA expression in rats, which is similar to the action of the GR antagonist, mifepristone [176]. While these studies have demonstrated that CHM constituents or formulas exert antidepressant-like activities through their actions on GR, the molecular mechanisms and specific acting proteins are still poorly understood. 

## 5. CHM Effects on the Neuroendocrine-Immune Network

As mentioned above, either the neuroimmune or neuroendocrine system plays a pivotal role in the pathogenesis of depression, but neither of these individual systems is fully responsible for the pathogenesis of depression. Indeed, clinical studies have demonstrated that abnormal neuroinflammatory responses of the immune system and dysfunction of the HPA axis commonly co-occur in depressive patients [177]. In addition, preclinical evidence has suggested that crosstalk exists between two biological systems through neural, endocrinal, and immunological interactions in the pathogenesis of depression (Figure 1).

Stress activates the HPA axis and sympathetic nervous system, resulting in neuroendocrinal and immunological changes, which, in turn, promote detrimental neuroinflammatory reactions [7,177,178,179,180,181]. Glucocorticoid immunomodulatory action is a key interaction between the HPA axis and neuroimmune system, which allows for coping with any situation that could challenge homoeostasis in the pathogenesis of depression [182,183]. Specifically, glucocorticoids exert immunomodulatory effects, primarily through GR-mediated inflammatory factors, including NF-κB and activator protein-1 [184,185,186]. Meanwhile, proinflammatory cytokines can also regulate the HPA axis by disturbing the GR function mediated by inflammatory signaling components, such as p38MAPK, NF-κB, and cyclooxygenase-2 (COX2) [8,47]. All MAPKs are potential targets of the anti-inflammatory actions of glucocorticoids through the inhibition of their phosphorylation, whereas proinflammatory cytokines induce the abnormal activation of MAPK signaling, which results in the alternation of GR phosphorylation and activity [8]. Furthermore, a chronic blockade of GR reverses GR dysfunction and decreases depressive-like behaviors induced by LPS [187,188]. 

Additionally, the activity of the HPA axis is also regulated by proinflammatory cytokines, such as IL-6, IL-1β, and TNF-α, which can easily cross BBB and exert their effects through various cytokine receptors [9]. In Li’s study, elevated CORT levels were observed in the plasma and hippocampus after the administration of LPS [189]. It has also been indicated that an intraperitoneal injection of IL-1 administered to rats activated the HPA axis by increasing the ACTH and corticosterone levels in plasma [190]. On the other hand, it has been demonstrated that the levels of TNF-α and IL-6 were upregulated by an intraperitoneal injection of CRF [191]. It is noteworthy that neuroinflammation in stress-induced animal models can be attenuated by the CRF_1_ antagonist, SSR125543 [192]. These findings have suggested that the release of CORT, ACTH, and CRF can be induced by proinflammatory cytokines and, conversely, proinflammatory cytokines can also be regulated by the modulation of HPA axis hormones. 

The HPA axis has been shown to be involved in microglial activation. Both CRF receptors and GR are abundantly distributed in microglial cells [193,194,195], and CRF stimulates release of TNF-α in cultured microglial cells [196]. High levels of glucocorticoids have been shown to participate in both proinflammatory cytokine production and the sensitization of microglial cells [6,197]. In addition, glucocorticoids induce microglial proliferation in restraint stress-induced mice [197]. However, due to a lack of correlation between the HPA axis and immune measures, the specific function of the HPA axis in microglial physiology and the mechanism by which chronic cytokine exposure influences the HPA axis function remains to be uncovered [198,199]. Overall, these studies have indicated that the reciprocal regulation between the HPA axis and neuroimmune system represents a common feature in the pathogenesis of depression.

**Figure 4:**
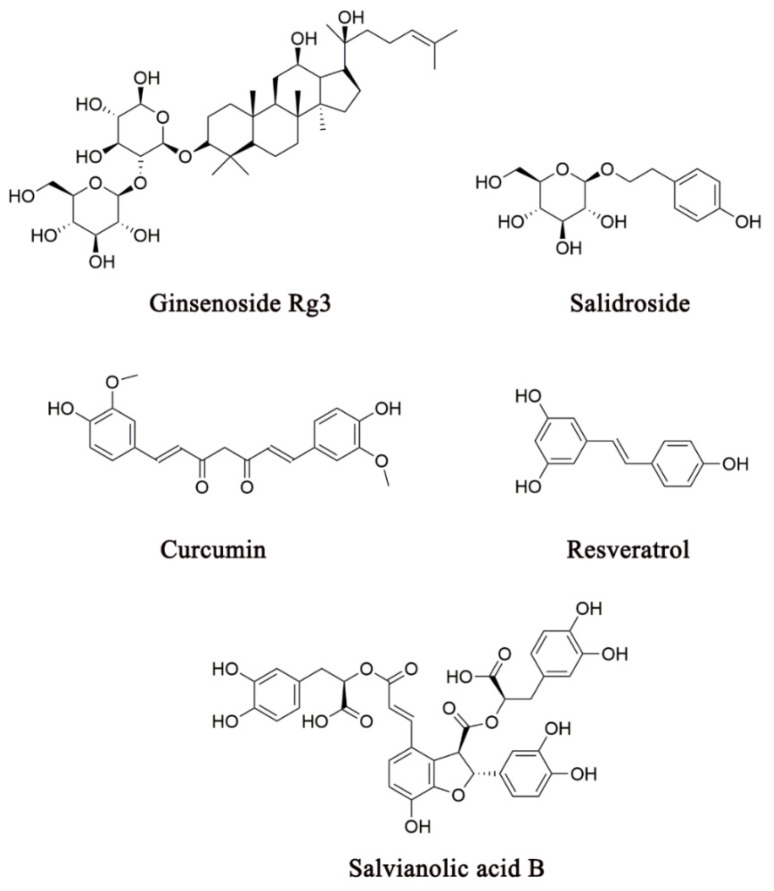
Several representative CHM constituents that have been shown to produce antidepressant effects through their actions on the neuroendocrine-immune network.

It has often been reported that a CHM herbal constituent exhibits multiple effects in the pathogenesis of depression [200,201,202]. Several representative CHM constituents that have been shown to exert multiple actions on the neuroendocrine-immune network are shown in Figure 4. Ginsenoside Rg3 (20 or 40 mg/kg, 3 days, i.g.) was isolated from *Panax ginseng* C.A. Meyer has been shown to effectively suppress LPS-induced neuroinflammation by reducing the proinflammatory cytokines (IL-1β, IL-6, and TNF-α), NF-κB signal pathway, and microglial activation in the brain [107]. It has also been reported that Rg3 (20 or 40 mg/kg, 14 days, i.g.) attenuated the hyperactivation of the HPA axis by reducing CRH, CORT, and ACTH in CUS rats [108]. Furthermore, it has been shown that total ginsenosides (200 mg/kg, 7 days, i.g.) significantly decrease serum CORT levels, increase GR mRNA expression, and reduce IL-1β, IL-6, TNF-α, and IDO in the hippocampus of LPS mice or CUMS rats [104,105].

Curcumin, a diarylheptanoid from *Curcuma longa* L., is another example of CHM constituents that possess multiple actions on the neuroendocrine-immune network. Xu et al. showed that curcumin (5 or 10 mg/kg, 21 days, p.o.) produces antidepressant activity by suppressing the aberrant activation of the HPA axis caused by an elevated serum CORT level and GR mRNA expression in CUS rats [116]. Interestingly, a recent study has shown that curcumin (100 mg/kg, 4 weeks, i.g.) significantly reduces the mRNA expression of proinflammatory cytokines, including IL-1β, IL-6, and TNF-α, and suppresses the activation of NF-κB signaling and the NLRP3 inflammasome in CUMS rats [115].

The phenomenon that one CHM constituent exerts multiple actions on several biological systems has been understood poorly because of the lack of experimental evidence to define its pharmacological profiles and specific interactions with its targeting proteins [12]. It is most likely the case that crosstalk exists between these biological systems or that one CHM constituent acts non-selectively on multiple targets [12]. This makes it difficult to understand the mechanisms of action of CHM constituents at the molecular level. Thus, more in-depth studies are required to uncover the specific interactions between these CHM constituents and their targeting proteins. Nevertheless, multi-target actions of these CHM constituents provide the scientific basis for interpreting their system-wide mechanisms of action.

In addition to these CHM constituents, many CHM antidepressant formulas have been shown to possess multiple underlying mechanisms of action, particularly on the HPA axis and neuroimmune system (Table 2). This is shown in the studies on Kai-Xin-San (KXS), an empirical antidepressant formula, which consists of *Panax ginseng* C.A. Meyer, *Poria cocos* (Schw.) Wolf, *Polygala tenuifolia* Willd, and *Acorus tatarinowii* Schott [203]. A chronic administration of KXS (338 or 676 mg/kg, 3 weeks, i.g.) has been shown to produce antidepressant-like activity in CUMS-induced animal models through the reduction of COX-2, IL-2, IL-6, and TNF-α expression levels and increase in IFN-γ and IL-10 production [29,138,204]. Notably, in other studies, KXS (0.9 or 2.7 g/kg, 5 weeks, i.g.) has also been reported to modulate the activity of the HPA axis by reversing the elevated ACTH level in CMS-induced mice [138,205]. Taken together, these studies indicate that the underlying mechanisms of KXS, as an antidepressant formula, include its actions on the neuroendocrine-immune network.

In comparison with single CHM herbal constituents, the multidrug feature of a CHM antidepressant formula confers its pharmacological actions on multiple targets toward diverse pathological systems. The antidepressant actions of KXS are triggered by its numerous bioactive constituents within the formula. For instance, ginsenosides Rg1, Rg3, Rh1, Rh3, Rb1, Rk1, and Rf from *Panax ginseng* C.A. Meyer have been demonstrated to exhibit dual actions against neuroinflammation and hyperactivation of the HPA axis [106,108,206,207,208,209,210,211,212,213,214,215], while 3,6’-disinapoyl sucrose and the oligosaccharide esters-enriched fraction, YZ50, from *Polygala tenuifolia* Willd have been shown to possess bioactivity that de-hyperactivates the HPA axis [216,217,218]. Additionally, poricoic acid A, isolated from *Poria cocos* (Schw.) Wolf, has been reported to produce anti-inflammatory effects by inhibiting prostaglandin E_2_ and NO production through a decrease in COX-2 and iNOS expression, respectively [219]. β-Asarone, a major bioactive constituent of *Acorus tatarinowii* Schott, has also been demonstrated to be an anti-inflammation agent, as it downregulates TNF-α, IL-1β, and IL-6 expression [220]. While the molecular interactions between these herbal constituents and their pharmacological targets remain to be uncovered, multiple actions of these constituents in KXS toward multiple biological systems, such as the neuroendocrine-immune network, represent an excellent example of CHM antidepressant formulas in the systematic treatment of depression. Likewise, many other CHM antidepressant formulas have been shown to possess multiple mechanisms of action on diverse biological systems, particularly the neuroendocrine-immune network, in the pathogenesis of depression (Table 2).

Dysfunction of the neuroimmune or neuroendocrine system results in profound effects on the CNS through the neuroendocrine-immune network. To uncover the system-wide mechanism of action of KXS, a study has been conducted to assess the protein expression in serum samples of depressive patients, before or after Shen-Zhi-Ling (a proprietary tablet formulated from KXS) treatment (3.2 g/day, 8 weeks, i.g.), using quantitative proteomic analysis [221]. Of a total of 878 serum proteins, the abnormal expression of 12 proteins in depressive patients could be reversed by treatment with KXS. Functional analysis further revealed that these proteins are implicated in platelet activation, immune regulation, and lipid metabolism. Moreover, a quantitative proteomic study has also been performed to evaluate the hippocampal proteins of CMS-induced rats in response to KXS administration (0.6 g/kg, 14 days, i.g.) [222]. This study identified 33 hippocampal proteins that are associated with KXS treatment. Protein–protein interaction network analysis showed that these proteins can be classified into several categories that participate in glutamate signaling, synaptic plasticity, the metabolic process, the cell survival process, and the BDNF, mTORC1, and cAMP pathways. These studies indicated that KXS exhibits antidepressant actions through targeting numerous proteins across multiple biological systems, providing a network or systems pharmacology approach to understanding the mechanism of action of KXS at the systems level.

## 6. Discussion

Numerous empirical CHM antidepressant formulas are often used in clinical practice for the treatment of depression (Table 2). To elucidate the mechanism of action by which a CHM formula exhibits antidepressant-like activity through the modulation of multiple biological factors across divergent systems is an important research direction. Two major pharmacological approaches are commonly employed in the analysis of the mechanism of action of a CHM formula on the biological factors in the pathogenesis of depression. One is the molecular approach, which uses single bioactive constituents from an herb used in a CHM antidepressant formula to explore their specific actions on potential pharmacological targets. The studies that employ the molecular approach have provided a scientific basis for revealing the mechanism of action of a CHM antidepressant herb or formula at the molecular level (Figure 2, Figure 3 and Figure 4 and Table 1). Because the constituent complexity and drug–drug interactions of an entire formula often prevent the molecular mechanism of action from being uncovered, the molecular approach plays a critical role in our understanding of the drug–target interactions in depression treatment. However, the effects of single molecular constituents cannot exactly reflect the action of a CHM composite formula, which contains numerous bioactive constituents that are proposed to simultaneously act on diverse pharmacological targets across biological systems. Hence, it is necessary to integrate the mechanism of action from the molecular level into the systems level in order to understand the role of CHM in depression treatment.

Another approach is the systems pharmacology approach, which involves uncovering the system-wide mechanism of action of an entire CHM antidepressant formula. Systems pharmacology studies drugs, drug targets, and drug effects at the systems level and reveals all responses to the pharmacological actions of drugs across various biological systems [223]. The systems pharmacology approach has recently been applied in studies of CHM antidepressant formulas and shown to be a powerful tool for understanding the system-wide mechanism of action (Table 2 and Section 5). It aims to create a network of the biological factors within a specific system or across diverse systems in response to the pharmacological actions of an entire CHM formula. Several advanced analysis techniques, including DNA or RNA microarray [200,224,225,226,227,228] and quantitative proteomics [229], have been used to identify the potential targeting proteins that are associated with a typical CHM formula. While systems pharmacology-based studies provide a holistic point of view on the pharmacological actions of a given CHM formula, they cannot provide detailed information on molecular drug–target interactions. In addition, the targeting protein candidates resulting from the system pharmacology-based analysis still require further validation by the molecular approaches. While these two approaches are commonly used in preclinical studies, neither can provide a holographic picture of the mechanism of action of a CHM formula in the treatment of depression. Therefore, it is vital to integrate the two approaches into the study of CHM in order to understand the mechanism of action of a CHM antidepressant formula in its entirety.

In summary, the neuroimmune or neuroendocrine system not only exhibit profound effects on the CNS, but also reciprocally regulate one another through the neuroendocrine-immune network. Thus, the effective approach to the treatment of depression induced by the dysfunction of the neuroendocrine-immune network should concurrently target multiple pathological factors across these biological systems. Preclinical studies have demonstrated that the holistic, multidrug, and multitarget CHM represents an excellent example of systems medicine in the treatment of depression. Therefore, we expect that CHM antidepressant formulation will be accepted broadly as an effective medication for the systematic treatment of depression.

## Data Availability

No new data were created or analyzed in this study. Data sharing is not applicable to this article.
